# Attenuation of temporal correlations of neuronal oscillations in patients with mild spastic diplegia

**DOI:** 10.1038/s41598-017-14879-8

**Published:** 2017-11-02

**Authors:** Fei Gao, Xiangci Wu, Yi Feng, Huibin Jia

**Affiliations:** 1 Department of Pain Medicine, Peking University People’s Hospital, Beijing, China; 2 Institute of Behavioral Psychology, Henan University, Kaifeng, Henan China; 3 Key Laboratory of Child Development and Learning Science of Ministry of Education, School of Biological Sciences & Medical Engineering, Southeast University, Nanjing, Jiangsu China

## Abstract

The aim of this study was to investigate the temporal correlations of neuronal oscillations in patients with mild spastic diplegia (MSD). Resting-state electroencephalography (EEG) was recorded from 15 male adolescent and young adult patients with MSD and 15 healthy controls. We characterized the temporal correlations of neuronal oscillations, both on long temporal scale (i.e., >1 second) and short-to-intermediate temporal scale (i.e., <≈1 second) using detrended fluctuation analysis (DFA) and an analysis of the life- and waiting-time statistics of oscillation bursts respectively. The DFA exponents at alpha and beta bands, the life-time biomarker of alpha oscillation, and the life- and waiting-time biomarkers of beta oscillation were significantly attenuated in the patients compared with controls. Moreover, altered scalp distributions of some temporal correlation measures were found at alpha and beta bands in these patients. All these findings suggest that MSD is associated with highly volatile neuronal states of alpha and beta oscillations on short-to-intermediate and much longer time scales, which may be related to cognitive dysfunction in patients with MSD.

## Introduction

Cerebral palsy (CP), which is a well-recognized neurodevelopmental disorder, is defined as “a group of permanent disorders of the development of movement and posture, causing activity limitation, that are attributed to non-progressive disturbances that occurred in the developing fetal or infant brain”^1^. Although CP is primarily a motor disorder, disturbances of sensation, perception, cognition, social communication and behavior often occur along with this disease^2^. The prevalence of CP is significantly influenced by the gestational age, with about 0.1% in children born at term and 14.6% in children born below 28 weeks gestational age^3^. Spastic diplegia is the most common type of CP as a result of injury to the periventricular white matter^4^, in which mild spastic diplegia (MSD) accounts for the majority in our clinical practice. In general, patients with MSD could walk independently, in spite of reduced balance, speed and coordination (i.e., they are at level I or II of Gross Motor Function Classification System [GMFCS])^5^.

Quantitative electroencephalography (EEG) has been widely used to investigate the pathophysiological underpinnings in patients with CP^6–8^. The most commonly and traditionally used measure in this field is the spectral power or amplitude of specific frequency band, which reflects the strength of locally synchronous cortical activation. However, inconsistent results have been reported in the literature, which may be caused by the age and clinical heterogeneity of patients with CP^6,7,9^. On the other hand, the EEG measures based on functional connectivity (e.g., coherence and spatial complexity), which could quantify the statistical dependence between spatially distributed synchronous neuronal assemblies, have also been used to identify the biomarkers that could discriminate patients with CP and healthy controls^6,7,10^. Typically, hypo- or hyper-connectivity for different EEG frequency bands has been detected in individuals with CP, which supports the theory that the disrupted cerebral connectivity in individuals with CP contributes to the social, cognitive, and behavioral phenotype^10^.

A major emphasis in the aforementioned studies is placed on the neuronal amplitudes and spatial neuronal interactions. However, the temporal structure of neuronal activity has been poorly addressed in CP. Microstate analysis is one of such methods capable of detecting the temporal organization of human EEG. Inspecting the time-course of EEG reveals that these electric field configurations change discontinuously, i.e. particular configurations remain quasi-stable for a duration of approximately 100 ms and then transition abruptly to another configuration^11^. The periods of quasi stability have been termed “microstates”. Cluster analytical approaches consistently extracted four head-surface brain electric field configurations, which were referred to as EEG microstate classes and were conventionally labeled from A through D^12^. Researchers found that the four EEG microstate classes each could explain one of the large-scale resting-state networks (RSNs) obtained from the blood oxygen level dependent (BOLD) signal^13^. Using resting-state EEG microstate analysis, Gao *et al*.^10^ revealed that the occurrence rate of microstate class A and D were significantly higher and the duration of microstate class B was significantly lower in the patients with MSD compared to healthy controls. This result suggests that MSD is associated with highly volatile neuronal states and higher temporal complexity of neuronal oscillations, thus it may be predicted that the temporal structure of neuronal oscillations in patients with MSD should be more random-like, even indistinguishable from a random process with no memory on long temporal scale (i.e., >1 second) and short-to-intermediate temporal scale (i.e., <≈1 second), which are especially relevant for sustained cognitive operations (e.g., memory coding, abstract reasoning and problem solving)^14–16^ and transient neural processes (e.g., vision, hearing and ache)^17–19^ respectively.

In the temporal scale from seconds to hundreds of seconds, the amplitude fluctuations of EEG or magnetoencephalography (MEG) oscillations, particularly for theta, alpha, and beta bands, are governed by power-law-form long-range temporal correlations (LRTCs), which indicates that the temporal auto-correlations attenuate very slowly in time dimension and has been confirmed in a dozens of studies using detrended fluctuation analysis (DFA)^20,21^. Previous works in computational models of neuronal oscillations revealed that LRTCs emerge only when neural networks produce critical neuronal avalanches and when excitatory and inhibitory connectivities are balanced^22,23^. Moreover, alterations of LRTCs have been observed in pre-clinical studies. For example, Nikulin *et al*.^20^ found that LRTCs were strongly attenuated in patients with schizophrenia for the amplitude envelopes of alpha and beta oscillations. In addition to LRTCs, the temporal structure of oscillations on short-to-intermediate time scales can be quantified using cumulative distribution functions of the life- and waiting-times of oscillation bursts. Previous research found that Alzheimer’s patients had a strongly reduced incidence of alpha-band oscillation bursts with long life- or waiting-times (<≈1  second) over temporo-parietal regions and markedly increased life- and waiting-times of theta oscillations over medial prefrontal regions^21^.

A growing body of findings suggests that the human brain operates near a critical regime^24,25^, which has been related to optimal information processing. Since the metastability of critical systems maximizes their dynamic range, storage capacity and computational power, any deviation from critical-state or loss of temporal correlations in neuronal oscillations on long temporal scale or short-to-intermediate temporal scale could result in significant cognitive deficiency.

Here, we measured resting-state EEG oscillations in the male adolescent and young adult patients with MSD and healthy controls. In the present study, we investigated the temporal correlations in neuronal oscillations on long temporal scale (i.e., >1 second) and short-to-intermediate temporal scale (i.e., <≈1 second). Since the presence of temporal correlations is related to optimal information processing, we hypothesized that LRTCs and incidence of long life- or waiting-times should be attenuated in patients with MSD. In addition, we also evaluated the relationship between the attenuated LRTCs and life- or waiting-time biomarkers in patients for each frequency band.

## Results

### Amplitude of neuronal oscillations

**Figure 1:**
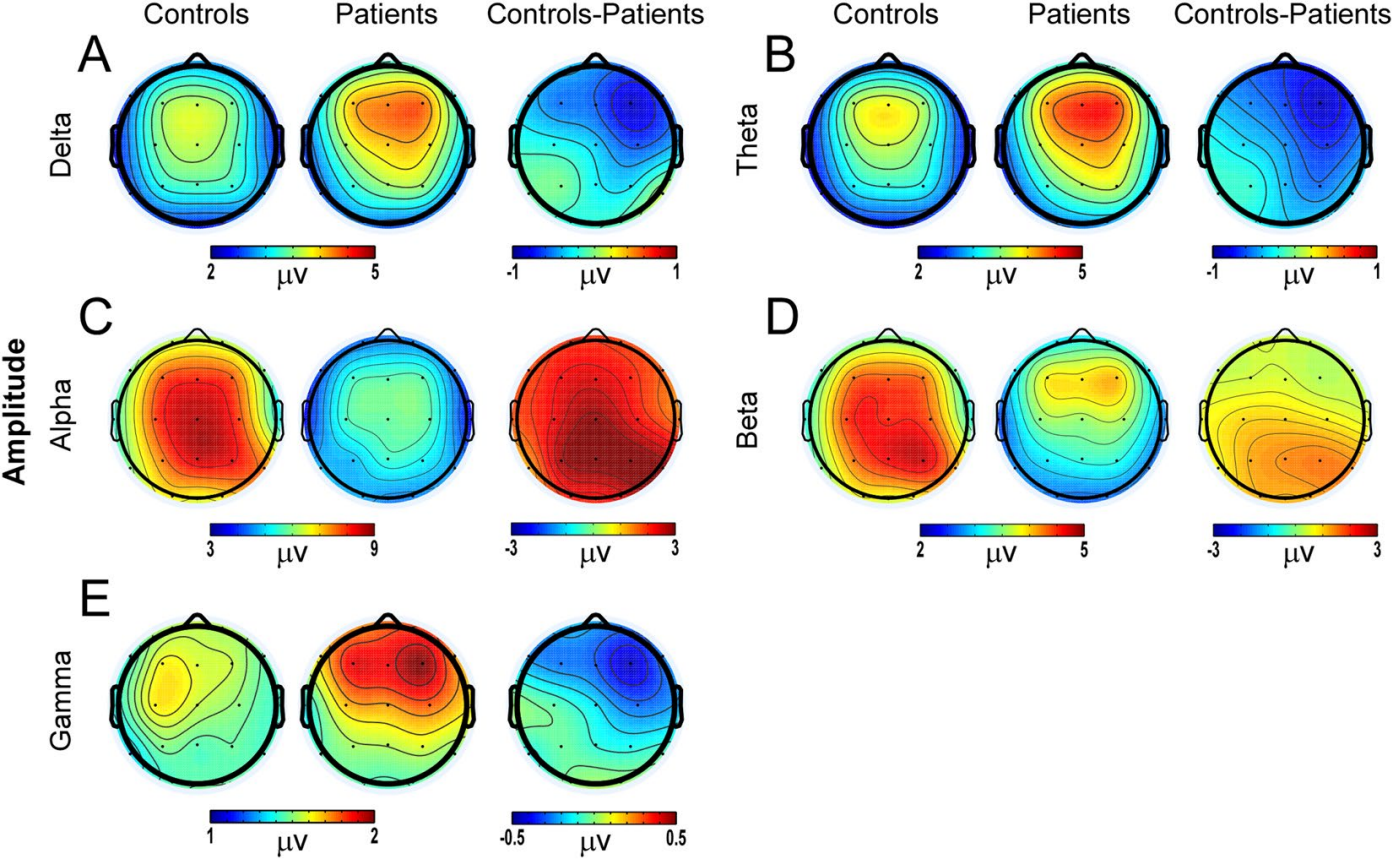
No significant amplitude group differences for delta, theta, alpha, beta, and gamma oscillations between the patients with MSD and healthy controls. For both groups, the spatial amplitude distributions of the delta, theta, and gamma oscillations showed the maximum amplitude over the fronto-central regions (Fig. 1A, B and E), while the alpha oscillation had the maximum amplitude over parieto-central areas (Fig. 1C). However, the scalp topography of amplitude of beta oscillation was maximum over parieto-occipital areas for healthy controls and over frontal areas for patients (Fig. 1D). The topographical randomization test on beta amplitude found that the map configuration of beta amplitude in patients was significantly different from that in healthy controls (*p* < 0.05).

The scalp topographies of amplitude are presented in Fig. 1. For both healthy controls and patients, the spatial amplitude distributions of the delta, theta, and gamma oscillations showed the maximum over the fronto-central regions (Fig. 1 A,B and E), while the alpha oscillation had the maximum amplitude over parieto-central regions (Fig. 1C). However, the scalp topography of amplitude of beta oscillation was maximum over parieto-central regions for healthy controls and over frontal regions for patients (Fig. 1D), which was confirmed by the topographical randomization test on beta amplitude (i.e., the map configuration of beta amplitude in patients was significantly different from that in healthy controls, *p* < 0.05). The topographical randomization tests on amplitude of the other four oscillations did not reach significance (*p* > 0.05).

For the amplitudes of all these five oscillations (i.e., delta, theta, alpha, beta, and gamma), the independent sample t-test conducted separately on each electrode did not reveal any significant results after the false discovery rate (FDR) procedure, although there is a clear trend that the alpha amplitudes of the healthy controls were larger than those of patients over parieto-occipital electrodes.

### DFA exponent of neuronal oscillations

**Figure 2:**
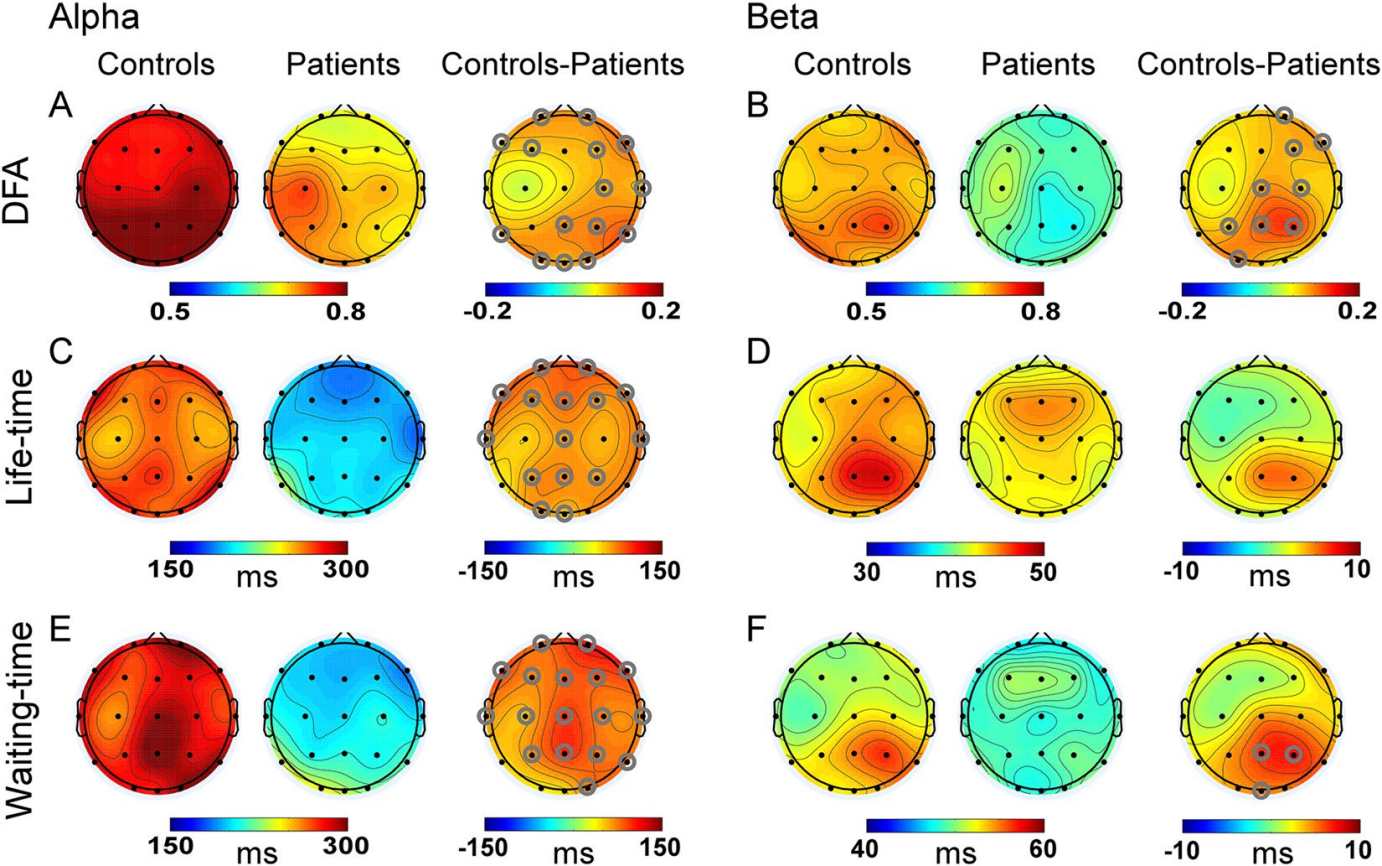
Aberrant temporal structure of alpha and beta oscillations in patients with MSD. Grand-average topographies of measures are shown for controls (left column), patients (middle column), and controls minus patients (right column). Alpha oscillations (8–12 Hz) are shown in the left panel (**A**, **C** and **E**) and beta oscillations (12–30 Hz) in the right panel (B, D and F). Grey circles denote channels with *p* < 0.05. Each row displays one measure: DFA exponent (**A**, **B**), life-time biomarker (**C**, **D**), waiting-time biomarker (**E**, **F**).

The scalp topographies of DFA exponent for alpha and beta oscillations are presented in Fig. 2A and B. The scalp topographies of the other three oscillations are not shown, since we did not detect any significant results on these oscillations. For healthy controls, the spatial distribution of the DFA exponent of alpha oscillation was similar to that of beta oscillation, showing largest DFA exponent over the parieto-occipital regions. However, the spatial distributions of the DFA exponent of alpha and beta oscillations were found maximum over left temporo-parietal regions in our patients. The topographical randomization tests on DFA exponent revealed that the map configuration of beta DFA exponent in patients was significantly different from that in healthy controls (*p* < 0.05). Significant group difference was not found for the map configuration of alpha DFA exponent.

For the DFA exponent of alpha and beta oscillations, the independent sample t-test conducted on each electrode revealed significant group differences in most scalp electrodes after the FDR procedure (i.e., the DFA exponent of alpha and beta oscillations in healthy controls were significant larger than those in patients). Significant group differences were not detected for the other three oscillations (i.e., delta, theta, and gamma).

### Life- and waiting-time biomarkers of neuronal oscillations

Figure 2C–F show the spatial distributions of the life- and waiting-time biomarkers for alpha and beta oscillations of both groups. The life- and waiting-time biomarkers’ scalp topographies of the other three oscillations are not presented in Fig. 2, since significant results were not found on these oscillations. For healthy controls, the spatial distributions of the life- and waiting-time biomarkers of alpha oscillation were similar to those of beta oscillation (i.e., the life- and waiting-time biomarkers were longest in the parieto-occipital electrodes, although this trend was less obvious for the life-time biomarker of alpha oscillation). However, the spatial distributions of the life- and waiting-time biomarkers of alpha oscillation were distinct to those of beta oscillation for patients. The life- and waiting-time biomarkers of alpha oscillation were shortest over the frontal regions, while those of beta oscillation were longest in the frontal electrodes. The topographical randomization tests on the life- and waiting-time biomarkers revealed that the map configurations of alpha waiting-time biomarker, beta life-time biomarker, and beta waiting-time biomarker in patients were significantly different from those in healthy controls (*p* < 0.05). Significant group difference was not found for the map configuration of alpha life-time biomarker.

For the life- and waiting-time biomarkers of alpha oscillation, the electrode by electrode independent sample t-test detected significant group differences in most scalp electrodes (i.e., the life- and waiting-time biomarkers of alpha oscillation in controls were significant longer than those in patients). However, for the waiting-time biomarker of beta oscillation, significant group effect only found in parieto-occipital electrodes (i.e., the waiting-time biomarker of beta oscillation in controls were significant longer than those in patients). Significant group difference was not found for the life-time biomarker of beta oscillation.

### Correlations between DFA exponents and life- and waiting-time biomarkers

**Figure 3:**
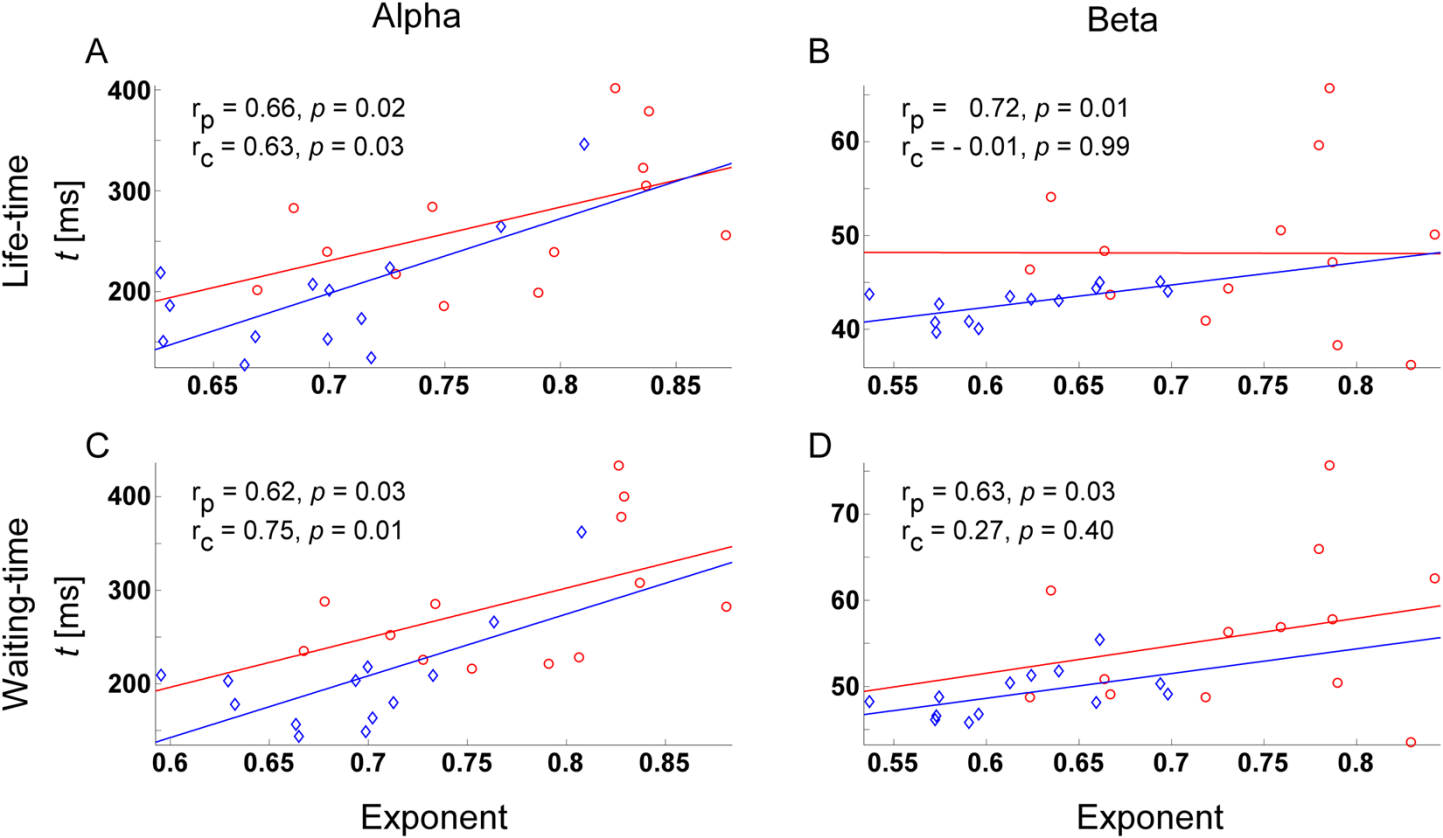
Autocorrelations on long time scales are related to the short-to-intermediate time-scale burst structure. Scatter plots of DFA exponent vs. life-time (**A**, **B**) and waiting-time (**C**, **D**) indices for patients with MSD (blue diamonds) and healthy controls (red circles) in the alpha (**A**, **C**) and beta (**B**, **D**) oscillations. Each value represents the average across channels with a significant group difference for both measures. Note that, since significant group difference was not found for life-time of beta oscillations, the average value in Fig. 3B was made across the 4 channels with the largest group difference both for DFA exponent and life-time. p, patients; c, controls.

Figure 3 shows the scatter plots of DFA exponents vs. life-time (A, B) and waiting-time biomarkers (C, D) for patients with MSD (blue diamonds) and healthy controls (red circles) of the alpha (A, C) and beta (B, D) oscillations over the electrodes with a significant group difference for both measures. The analysis over correlation coefficients, estimated using the average across electrodes with a significant group difference for both measures, found that for patients, (1) the DFA exponent of alpha oscillation was significantly correlated with the life- and waiting-time biomarkers of alpha oscillation (*r* = 0.66, *p* = 0.02 < 0.05 and *r* = 0.62, *p* = 0.03 < 0.05 respectively), (2) the DFA exponent of beta oscillation was significantly correlated with the life- and waiting-time biomarker of beta oscillation (*r* = 0.72, *p* = 0.01 < 0.05 and *r* = 0.63, *p* = 0.03 < 0.05 respectively). For healthy controls, the DFA exponents of alpha oscillations was significantly correlated with the life- and waiting-time biomarkers of alpha oscillations (*r* = 0.63, *p* = 0.03 < 0.05 and *r* = 0.75, *p* = 0.01 < 0.05 respectively), but the DFA exponents of beta oscillations was not significantly correlated with the life- and waiting-time biomarkers of beta oscillations (*r* = −0.01, *p* = 0.99 > 0.05 and *r* = 0.27, *p* = 0.40 > 0.05 respectively). In addition, we have tested whether there were significant group effects for these correlation coefficients, and found that the group effect of correlation coefficient between the DFA exponent and the life-time biomarker was significant for beta oscillations (*p* = 0.04 < 0.05).

**Figure 4:**
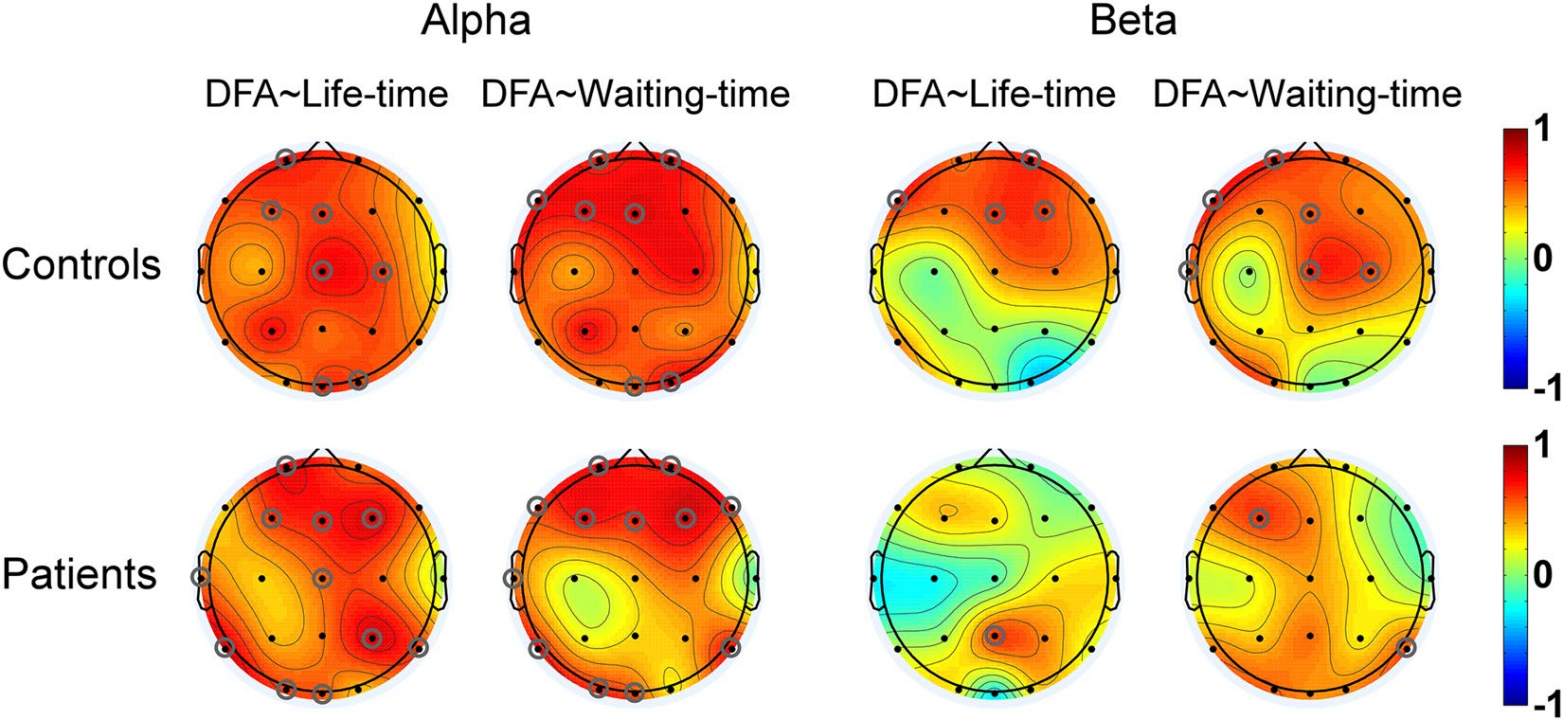
The topographies of correlation coefficients between the DFA exponent and the life- and waiting-time biomarkers for alpha and beta oscillations in the patients and controls. Grey circles denote the channels with significant correlations between the DFA exponent and the life- and waiting-time biomarkers (*p* < 0.05). DFA~Life-time: the correlation between the DFA exponent and life-time biomarker. DFA~Waiting-time: the correlation between the DFA exponent and waiting-time biomarker.

An electrode-by-electrode analysis of correlation coefficients between DFA exponent and the life- and waiting-time biomarkers for alpha and beta oscillations were also conducted, and the results were shown in Fig. 4. For each frequency band and each electrode, statistic tests did not reveal any significant group effect for these correlation coefficients.

## Discussion

In this study, we measured ongoing neuronal oscillations with multi-channel EEG at resting-state and eyes-closed condition in the patients with MSD and healthy controls, and quantified the temporal correlations of neuronal oscillations (i.e., delta, theta, alpha, beta, and gamma) on long temporal scale (i.e., >1 second) and short-to-intermediate temporal scale (i.e., <≈1 second). Although significant group differences were not observed for the amplitudes of these oscillations, strongly attenuated LRTCs and short-to-intermediate temporal correlations of alpha and beta oscillations were identified in the patients compared with healthy controls. Using topographical randomization tests, we found that the map configurations of specific measures in patients (i.e., the waiting-time biomarker of alpha oscillation, the amplitude of beta oscillation, the DFA exponent of beta oscillation, and life- and waiting-time biomarkers of beta oscillation) were significantly different from those in healthy controls. These results suggested that the neural circuits responsible for generation of neuronal oscillations and the coordination of neuronal processes in time may have been affected in our patients. We also found that the DFA exponent was significantly correlated with the life- and waiting-time biomarkers for alpha and beta oscillation in the patients, indicating that the two types of aberrant temporal autocorrelations may share similar pathophysiological mechanisms in these patients.

### LRTCs in the patients with MSD

Long-range temporal correlations (LRTCs) indicate that events in the past affect the development of the process in the future^26^. The analysis of LRTCs provides a quantitative index of statistical dependencies in neuronal oscillations over different time scales^27^. One feature of LRTCs is its power-law behavior, suggesting that the underlying neuronal dynamics are similar on different time scales^26^. The presence of LRTCs in neuronal oscillations is thought to be beneficial for a reliable transfer of information in neuronal networks^28^. The stability of oscillations or their slow amplitude modulation may be critical for cognitive processes that require coordination of temporally distributed activity on time scales of several seconds^27^. Smit *et al*.^29^ speculated that the strength of LRTCs is the temporal integration span of brain activity and its information content, which is crucial for cognitive function of the brain. Strong LRTCs correspond to larger integration times^30^, which indicates the strong ability to maintain transiently stable oscillations in support of active neuronal representations during sustained cognitive operations (e.g., memory coding, abstract reasoning and problem solving).

In this study, significant group differences were not observed for the amplitudes of neuronal oscillations (i.e., delta, theta, alpha, beta, and gamma bands). Although significant group effects on the amplitudes or power of EEG oscillations have been observed in other studies, inconsistent results have been reported^6,9^, which may be caused by the age and clinical heterogeneity of patients with CP. On the other hand, topographical randomization test revealed the map configuration of beta oscillation amplitude has been altered in our patients with MSD. The beta oscillation amplitude is maximum over parieto-occipital areas and frontal areas for healthy controls and patients respectively. This result suggested that the neural circuits responsible for beta oscillation’s generation may have been altered, although its overall activity remained unchanged.

Using detrended fluctuation analysis, the present study revealed markedly weak LRTCs for alpha and beta oscillations under resting-state condition over most scalp electrodes in the patients with MSD, which can be hypothesized to underlie an excessive switching between the neuronal states in these patients. Note that, we found the DFA exponents of all the five oscillations, all the electrodes and all the patients were greater than 0.5 and less than 1.0, which indicated that although the DFA exponents of alpha and beta oscillations were attenuated in these patients, EEG oscillations in both the patients with MSD and healthy controls exhibited LRTCs with power-law behavior.

Moreover, the spatial distribution of DFA exponent for beta oscillation in the patients was significantly distinct from that in the controls, indicating that the neural network associated with DFA exponent for beta oscillation may be altered in the pathological condition. For healthy controls, the topography of DFA exponents was comparable for alpha and beta oscillations, showing largest scaling exponents in the parietal and occipital areas, which was consistent with previous studies^20^. For patients with MSD, lower similarity between the topography of DFA exponents of alpha band and that of beta band could be observed. In addition, this topography of DFA exponents was similar to that of amplitude distribution for healthy controls. Observing the spatial distributions of DFA exponents and amplitudes for alpha and beta oscillations of both groups, we found that (1) the LRTCs of alpha oscillation were stronger than those of beta oscillation; (2) the spatial homogeneity across scalp electrodes of DFA exponents was greater than that of amplitude, which may be partly caused by the volume conduction. These characters of DFA exponents have also been found in other studies^20^.

The presence of LRTCs indicates that the human brain operates with self-organized criticality (SOC), i.e., the neuronal oscillations display a spatial and temporal scale-invariance characteristic of the critical point of a phase transition, but without the need to tune control parameters to a precise value^24^. The SOC and LRTCs were advocated as being crucial to the information transmission of neuronal system^28,31^. Critical state might represent an optimal compromise for the competing demands of stability and information transmission in the neuronal networks^28^. Thus the decreased DFA exponents and its aberrant topographic distributions for alpha and beta oscillations may indicate decreased efficiency of brain information processing and impaired brain functions in our patients with MSD.

### Short-to-intermediate temporal correlations in the patients with MSD

The avalanche analysis originally from the study of neuronal avalanches, a phenomenon involves a spatial dimension and time scales much shorter than those of LRTCs (<≈1 second vs. >1 second, respectively), was adopted to characterize the temporal structure of neuronal oscillations on short-to-intermediate temporal scale. The findings in our study support our hypothesis that the amplitude modulation of local oscillatory activity holds information about pathophysiological changes in our patients with MSD.

Similar to the approach in Montez *et al*.^21^, the time periods that neuronal oscillations were above and below the median value of instantaneous amplitude were termed as life- and waiting-times, respectively. Then, the 95^th^ percentile of the cumulative probability distributions of life- and waiting-times were extracted and defined as “life- and waiting-time biomarkers”, which serve as measures of the probability of oscillations to have long life- or waiting-times in a given electrode location and frequency band. Combining the DFA exponents and life- and waiting-time indices can provide us more comprehensive information about the temporal autocorrelations of neuronal oscillations.

Using the above approach, we found that compared with healthy controls, the patients with MSD had a strongly reduced incidence of alpha-band oscillation bursts with long life- or waiting-times (<1  second) over most scalp regions and markedly reduced probability of beta-band oscillation bursts with long waiting-times (<1  second) over parieto-occipital areas. The results indicate a more random autocorrelation structure for alpha and beta oscillations on short-to-intermediate time scale in these patients.

The map configurations of alpha waiting-time biomarker, beta life-time biomarker, and beta waiting-time biomarker in patients were significantly different from those in healthy controls (*p* < 0.05). Note that, the map configurations of beta DFA exponents and amplitudes also show significant group effect. Thus, all the measures used in the present study (i.e., amplitude, DFA exponent, and the life- and waiting-time biomarkers) show significant map configuration differences on beta oscillation. On the other hand, for alpha oscillation, significant map configuration difference only detected on the waiting-time biomarker. The reduction of incidence of beta-band oscillation bursts with long life- or waiting-times was more likely caused by the altered neural network of life- or waiting-times for beta oscillation in this pathological condition.

### General discussion on the altered temporal autocorrelations in the patients with MSD

In recent years, increasing evidence suggests that scale-free brain activity contributes actively to brain functioning^32^. The amplitude fluctuations of narrow-band brain oscillations in EEG recordings exhibit the property of scale-free dynamics^33^. As mentioned above, although the amplitudes of oscillations were not affected in our patients with MSD, certain measures of alpha and beta bands derived from temporal autocorrelation analysis and their scalp distributions were found altered in these patients, which indicated that the amplitude modulation of brain oscillatory activity at rest holds information about pathophysiological changes in the patients with MSD. These findings support our hypotheses that the temporal structure of neuronal oscillations in patients with MSD should be more random-like on long temporal scale (i.e., >1 second) and short-to-intermediate temporal scale (i.e., <≈1 second), and are consistent with our recent study which identified that MSD is associated with highly volatile neuronal states of neuronal oscillation using resting-state EEG microstate analysis technique^10^. In line with our findings, Sajedi *et al*.^9^ also found higher complexity of brain dynamics in resting-state EEG in patients with CP, indicating that the CP brains’ neuronal network functions more randomly and irregularly than a normal brain.

The alpha and beta oscillations of EEG signals are considered to reflect cortical functions^8^, and may be closely associated with cognitive process. Alpha oscillations were demonstrated to reflect active inhibition of task irrelevant neuronal process^34^, and they may reflect segregation of neuronal processes involved in cognition^35^. And alpha oscillations in the occipito-parietal and central areas have similar functional significance related to attention and inhibitory processes^35,36^. Beta oscillations may relate primarily to the maintenance of the current sensorimotor or cognitive state^37^. Previous literature shows altered spectral power and functional connectivity in alpha and beta bands in patients with CP. For example, Kulak *et al*.^8^ found significantly lower alpha and beta powers and decreased interhemispheric coherence values in alpha and beta bands in patients with CP compared with healthy controls. Sajedi *et al*.^9^ found lower alpha power of resting-state EEG in patients with CP than normal controls. Attenuated alpha and beta LRTC would indicate a potential for high probability of switching between the mental states, and may be the neural basis of decreased brain functions (e.g., attention, and motor control), which are impaired in patients with MSD.

Moreover, strong linear correlations were detected between the DFA exponents of alpha and beta oscillations and the life- and waiting-time biomarkers of alpha and beta oscillations in patients. These results indicate that (1) temporal correlations on short-to-intermediate time scales could extend to much longer time scales; (2) the aberrant short-to-intermediate and long-range temporal correlation in the patients with MSD may share some common pathophysiological mechanisms.

### Limitations of the present study

As far as we know, this was the first study to investigate the temporal structures of neuronal oscillations in patients with MSD, even with CP. Several important and interesting findings have been revealed in this study, which suggest that the temporal correlations, both on long temporal scale (i.e., >1 second) and short-to-intermediate temporal scale (i.e., <≈1 second), were attenuated in patients with MSD. However, several limitations need to be mentioned. Firstly, standard neuropsychological and cognitive scales were not used to assess the psychological and cognitive functions of these patients. In previous study, using part of these patients, we found that their visual categorization ability were impaired although they had normal or corrected normal vision acuity and hardly any strabismus and nystagmus, without severe vision impairment^38^. However, due to the lack of objective assessments of cognitive abilities in the patients, the associations between attenuated temporal correlations and cognitive deficiency were not very clear. Secondly, the temporal correlations computation of this study is based on the dynamics of amplitude envelope of filtered scalp EEG signals, which has poor spatial resolution. Moreover, the scalp signals could be considered as mixtures of many source signals in the brain. Mixing of these source signals may alter the temporal correlations of neuronal oscillations. Spurious effects could arise when MSD is associated with increased mixing of sources due to many reasons (e.g., the structural differences in their brains, shorter spatial distances between different sources, and increased number of sources), even if the temporal structures of these source signals were similar between the two groups. Studying the dynamics of amplitude envelope of source-level signals could partly solve these limitations. However, since the total number of scalp electrodes in this study is only 20, the source localization precision of EEG data in this study should be very low.

## Conclusions

In the present study, the temporal correlations of neuronal oscillations of alpha and beta band, both on long temporal scale (i.e., >1 second) and short-to-intermediate temporal scale (i.e., <≈1 second), were attenuated in the patients with MSD, although the amplitudes of all the five oscillations (i.e., delta, theta, alpha, beta, and gamma) were not found to be significantly altered. In addition, using topographical randomization tests, we found that (1) for beta oscillation, all the measures used in the present study (i.e., amplitude, DFA exponent, and the life- and waiting-time biomarkers) show significant map configuration difference; (2) for alpha oscillation, significant map configuration difference only detected on the waiting-time biomarker. Lastly, linear correlations were detected between the DFA exponents and the life- and waiting-time biomarkers for alpha and beta oscillations in these patients. The aberrant temporal correlations on neuronal oscillations may be related to cognitive deficiency in patients with MSD.

All these findings suggest that MSD is associated with highly volatile neuronal states of neuronal oscillation on short-to-intermediate time scales and much longer time scales, which may be the starting point of observed cognitive deficiency in patients with MSD. The temporal structure of brain activity is at least as important as the amplitude or power of the neuronal oscillations as a measure of pathophysiology in MSD.

## Methods

### Participants

All participants were informed about the purpose of the present study before consenting to participate. All the participants and their parents signed an informed consent form for this experiment, which was approved by the Medical Ethic Committee of Beijing Dongzhimen Hospital and conducted in accordance with the principles of the Declaration of Helsinki.

The cases consisted of 15 right-handed male adolescent and young adult patients with MSD, the age of which was 16.5 ± 2.6 years (mean ± standard deviation). All the patients were right-handed according to the Edinburgh Handedness Inventory^39^. Since there are sex differences in the white matter structures of the adult brain^40^, only male patients were chosen in this study. More detailed information about these patients can be seen in Gao *et al*.^10^.

The control group consisted of 15 right-handed healthy male adolescent and young adult controls, the age of which was 18.3 ± 2.8 years. The patients and controls did not differ significantly with respect to age (*p* > 0.05). None of the controls has history of neurological or psychiatric disorders.

### EEG recording

During the experiment, all the participants seated on a comfortable chair in a silent, temperature-controlled room, and were instructed to keep relaxed and eyes closed. Every minute they were asked to report their conscious state, in order to prevent them from becoming drowsy during the eyes-closed condition. This eyes-closed period lasted for about 5 min.

Five minutes eyes-closed resting-state EEG data were recorded using 20 Ag/AgCl scalp electrodes (i.e., Fp1, Fp2, F7, F3, Fz, F4, F8, T3, C3, Cz, C4, T4, T5, P3, Pz, P4, T6, O1, Oz and O2) according to the international 10–20 system (ASA-Lab, ANT B.V., Netherlands). EEG data were referenced to the average of both mastoids. All electrode impedances were kept lower than 10 kΩ. The signals were recorded in the 0.16–100 Hz frequency range and sampled at 256 Hz.

### EEG data preprocessing

The EEG data recorded above were pre-processed using EEGLAB^41^, an open source toolbox running in the MATLAB environment, and in-house MATLAB functions. The continuous EEG signals were broadband filtered 0.5~80 Hz followed by the Blind Source Separation (BSS)^42^. Components reflecting eye movements, electromyography (EMG) or any other non-physiological artifacts were identified and removed. Since most patients failed to keep eyes closed without any large body movements for 5 minutes, only EEG data of the first two minutes of all participants were used in subsequent analyses. It should be noted that the EEG data of two patients and two healthy controls were excluded from further analysis due to extremely high noise.

### Extraction of instantaneous amplitude of neuronal oscillations

The subsequent analyses of the present study are based on the instantaneous amplitude (i.e., amplitude envelope) of neuronal oscillations, which was extracted using band-pass filters and the Hilbert transform.

Firstly, the pre-processed EEG data were band-pass filtered with finite impulse response filters (FIR) in order to extract the neuronal oscillations of the following five bands: delta (2–4 Hz), theta (4–7 Hz), alpha (8–12 Hz), beta (12–30 Hz), and gamma (30–45 Hz). In order to avoid introducing long-range correlations in neuronal oscillations, we used finite impulse response filters (FIR), instead of infinite impulse response filters (IIR). The order of the FIR filters was chosen so that it included three cycles of the low-frequency component of the band considered (i.e., 384 for delta, 192 for theta, 96 for alpha, 64 for beta, and 25 for gamma), in order to accurately detect the oscillations while also limiting the temporal integration caused by FIR.

Secondly, the instantaneous amplitude (i.e., amplitude envelope) of the band-pass filtered signals was extracted through the Hilbert transform. The band-pass filtered signals should not be directly used in the following temporal correlation analyses, since the band-pass filtered signals will appear as strongly anti-correlated signals in detrended fluctuation analysis (DFA).

For band-pass filtered signal *Y*(*t*), we can obtain its analytic signal *Y*_*an*_(*t*) through the Hilbert transform, as

1 $${Y}_{an}(t)=Y(t)+i{Y}_{H}(t),$$

where *Y*_*H*_(*t*) is the Hilbert transform of signal *Y*(*t*). Namely:

2 $${Y}_{H}(t)=\frac{1}{\pi }P.V.{\int }_{-\infty }^{+\infty }\frac{x(\tau )}{t\,-\,\tau }d\tau ,$$

where *P*.*V*. is the Cauchy’s Principal Value. Then, the instantaneous amplitude (i.e., amplitude envelope) of *Y*(*t*) is calculated as

3 $$A(t)=\sqrt{Y{(t)}^{2}+{Y}_{H}{(t)}^{2}}.$$

For each scalp electrode and each participant, the mean oscillation amplitude of each frequency band was computed as the time-averaged instantaneous amplitude (i.e., amplitude envelope).

### Detrended fluctuation analysis (DFA)

Neuronal oscillations exhibit scale-free amplitude modulation as reflected in power-law decay of autocorrelations, which is known as “long-range temporal correlations” (LRTCs). Although it could be assessed through the autocorrelation function or power spectrum of signals, the detrended fluctuation analysis (DFA), proposed by Peng *et al*.^43^, has proven to be a particularly robust method for studying the LRTCs in human physiological signals (e.g., EEG, MEG, functional magnetic resonance  imaging), since it is insensitive to the nonstationarity of neurophysiological processes^20,21,43,44^. As has been illustrated in Hardstone *et al*.^45^, the procedures of DFA for each frequency band, each scalp electrode and each participant can be summarized as follows:

1. Assuming *A*(*t*) was the amplitude envelope of neuronal oscillations extracted with the Hilbert transform at time t, we could compute the cumulative sum of *A*(*t*) to create the signal profile:
  4 $$X(t)=\sum _{k=1}^{t}A(k)\,-\,\langle A\rangle ,$$
  where <*A*> is the mean of the time series. This operation could eliminate the global trend of the amplitude envelope. Moreover, the signal profile *X*(*t*) makes less assumption about the stationarity of neurophysiological signals than the amplitude envelope *A*(*t*).
2. We defined a set of window lengths, T, which were equidistantly distributed on a logarithmic scale between 1 to 15 seconds. For each window length *τ* ∈ T, the signal profile *X*(*t*) was divided into a set of signals with length *τ* and 50% overlapping. The standard deviation of each signal with length *τ* was computed after its linear trend has been removed through a least-squares fit. Then the fluctuation function for window length *τ*, $\langle F(\tau )\rangle$, was computed as the mean standard deviation of signal with length *τ*.
3. After the fluctuation functions $\langle F(\tau )\rangle$ of all window length have been computed, we plotted the fluctuation function for all window lengths on logarithmic axes. Usually the relationship between $\langle F(\tau )\rangle$ and τ has a linear form in a double logarithmic coordinate system across many sizes of τ. The slope of the least-squares line in this graph is called the DFA exponent, *α*, which could quantify LRTCs.

If 0 < *α* < 0.5, then the time series is anti-correlated (i.e., the fluctuations are smaller in larger time windows than expected by chance). If *α* ≈ 0.5, then the time series is a uncorrelated and random process with no LRTCs. If 0.5 < *α* < 1, then the time series is positive correlated (i.e., the fluctuations are larger in larger time windows than expected by chance). If 1 < *α* < 2, then the time series is non-stationary.

The DFA exponent is interpreted as an estimation of the Hurst parameter. If 0 < *α* < 1, the time series can be modeled by a fractional Gaussian noise (fGn) with the Hurst parameter *H* = *α*. If 1 < *α* < 2, the time series can be modeled by a fractional Brownian noise (fBn) with the Hurst parameter *H* = *α* − 1.

### Assessing the validity of DFA

In the above DFA, the power law behavior is characterized in terms of the linear scaling between the fluctuation functions and window lengths on logarithmic axes. However, we have no prior knowledge about whether these log-log plots were truly linear. The DFA exponents could be calculated as long as these fluctuation functions increase with window lengths. In this study, a maximum likelihood based model selection technique introduced in Botcharova *et al*.^46^ was used to determine whether a given DFA fluctuation plot was best-approximated by a linear model. Briefly speaking, this technique fits these log-log plots with a set of alternative models (i.e., polynomial, root, exponential, logarithmic and spline with 2–4 sections) and compares the fit of each model using the Akaike Information Criterion (AIC) and Bayesian Information Criterion (BIC)^47^. Only the DFA exponents computed from the log-log plots that were found to be linear were used in the statistical tests.

### Life- and waiting-times analysis of oscillation burst

The method to estimate the “life- and waiting-time biomarkers” has been illustrated and verified in Montez *et al*.^21^. Briefly, the procedures of life- and waiting-times analysis of oscillation burst for each frequency band, each scalp electrode and each participant can be summarized as follows: The median amplitude of instantaneous amplitude (i.e., amplitude envelope) was computed, which was used to define the beginning and the end of an oscillation burst. The periods of the amplitude envelope remaining above and below this median value were termed as life- and waiting-times, respectively. Usually, the probability distributions of “life-times” and “waiting-times” exhibited power-law-like decays, which could be indicated by the least-squares fit in the logarithmically transformed plot (Fig. S1)^21,48^. The 95^th^ percentile of the cumulative probability distributions was used as an index that captures the fat tail in the distribution of “life-times” and “waiting-times”, which was called the “life- and waiting-time biomarkers”^21^. Lower life- and waiting-time biomarkers indicated that the incidence of long neuronal bursts is lower, and the signal has a more random autocorrelation structure on short-to-intermediate time scales^21^.

### Statistical analysis

Firstly, for each scalp electrode and each frequency band, independent sample t-test was applied with group (patients with MSD and healthy controls) as independent variable and the measures calculated above, including amplitude, DFA exponent, and the life- and waiting-time biomarkers, as dependent variables. Besides, the age was included as a covariate. To control for multiple comparisons, the significance level (*p* value) was corrected using FDR procedure^49^.

Secondly, in order to quantify the relationship between temporal correlations of neuronal oscillations on long temporal scale and those on short-to-intermediate temporal scale, the Pearson correlation coefficients between DFA exponents and life- and waiting-time biomarkers were estimated using the average across electrodes with a significant group difference for both measures and the value of each scalp electrode after controlling for the variable *age*. The Pearson correlation coefficients were calculated for each group, respectively. The significance of the correlation coefficients was assessed with *t*-statistic.

Thirdly, for each frequency band and each measure calculated above, the map configurations over participants were compared between patients with MSD and healthy controls using topographical randomization test as in Koenig *et al*.^50^, which was performed in the following way: (1) The global map dissimilarity (GMD) between the group-averaged topographical maps was computed. GMD is an index to describe topographic differences between conditions regardless of the relative strength, and is obtained by calculating the square root of the mean squared differences between all corresponding electrodes after two given scalp topographies have been recalculated against the average reference and normalized to unitary strength (i.e., divided by its own global field power)^51^. The GMD index is inversely related to the spatial correlation between two scalp topographies and varies from 0 (map homogeneity) to 2 (map inversion)^52^. (2) The individual topographical maps of all the participants were randomly assigned to two groups, and the new group-averaged topographical maps were obtained through averaging the individual maps within each group. A new GMD was computed. This procedure repeated for 5,000 times, which yielded the GMD distribution under the null hypothesis. Chance probability of obtaining GMD larger or equal to the observed GMD was established^50^.

Finally, we systematically assessed the Pearson correlation coefficients between age and the metrics used here (i.e., DFA exponent, life-time biomarker and waiting-time biomarker) on each electrode, each group and each frequency band. Figure S2 illustrates the relationship between age and the metrics that reflected temporal correlations on the electrodes with the maximum correlation coefficient for alpha and beta band. Note that, although these correlation coefficients were significant on certain electrodes, they were not significant after FDR correction. Even so, the variable age was used as covariate anyway in the current study.

## Electronic supplementary material

supplementary information
