# Supplementary material for: Attenuation of temporal correlations of neuronal oscillations in patients with mild spastic diplegia
Source: Sci Rep. 2017 Nov 2;7:14966. doi: 10.1038/s41598-017-14879-8 (PMC5668314; doi:10.1038/s41598-017-14879-8)
Supplement: Supplementary file 1 — supplementary information [file 41598_2017_14879_MOESM1_ESM.pdf]

Attenuation of temporal correlations of neuronal oscillations in patients with mild spastic diplegia

Fei Gao[1] Xiangci Wu[2] Yi Feng[1]* Huibin Jia[3]*

Fei Gao and Xiangci Wu contributed equally to this work.

1. Department of Pain Medicine, Peking University People's Hospital, Beijing, China.
2. Institute of Behavioral Psychology, Henan University, Kaifeng, Henan, China.
3. Key Laboratory of Child Development and Learning Science of Ministry of Education, Research Center for Learning Science, School of Biological Sciences & Medical Engineering, Southeast University, Nanjing, Jiangsu, China.

*Correspondence to:

Yi Feng: Chair and Professor, Department of Pain Medicine, Peking University People's Hospital, No. 11 South Street Xizhimen, Xicheng District, Beijing, China.

E-mail: doctor_yifeng@sina.com

Huibin Jia: Key Laboratory of Child Development and Learning Science of Ministry of Education, Research Center for Learning Science, School of Biological Sciences & Medical Engineering, Southeast University, Nanjing, Jiangsu, China.

E-mail: huibin_jia@foxmail.com

Fig.S1

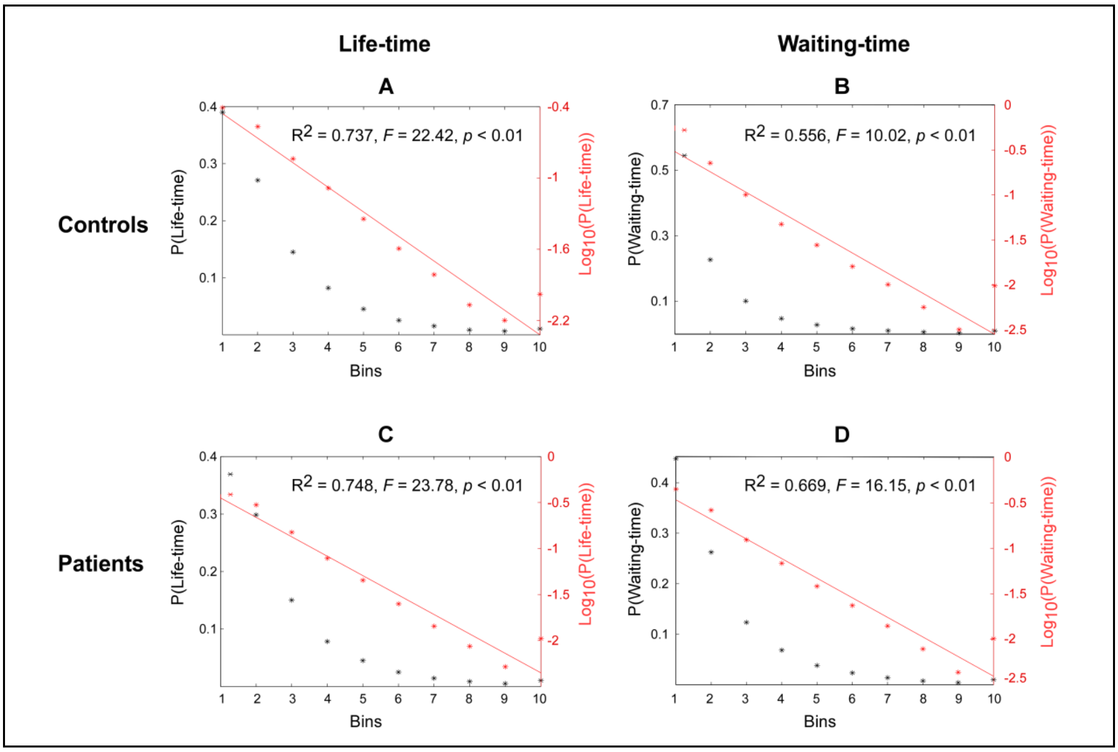

Fig.S1 The grand-averaged probability distributions of life-times and waiting-times for alpha band in healthy controls and patients. For each electrode and each subject, the life-times and waiting-times of alpha band were divided into 10 equal-width bins, and the probability in each bin was computed. Then, for the patient and control group respectively, the grand-averaged probability distributions of life-time and waiting-time across all electrodes and all subjects in each group were obtained (see black asterisks in panel A, B, C, and D). The grand-averaged probability distributions after logarithmic transformation were also computed (see red asterisks in panel A, B, C, and D). These logarithmically transformed probability distributions were least-squares fitted to straight lines (red line in panel A, B, C, and D). The statistical characteristics (i.e., R^2, p value) of these four linear models indicated that the original probability distributions exhibited power-law-like decays.

Fig.S2

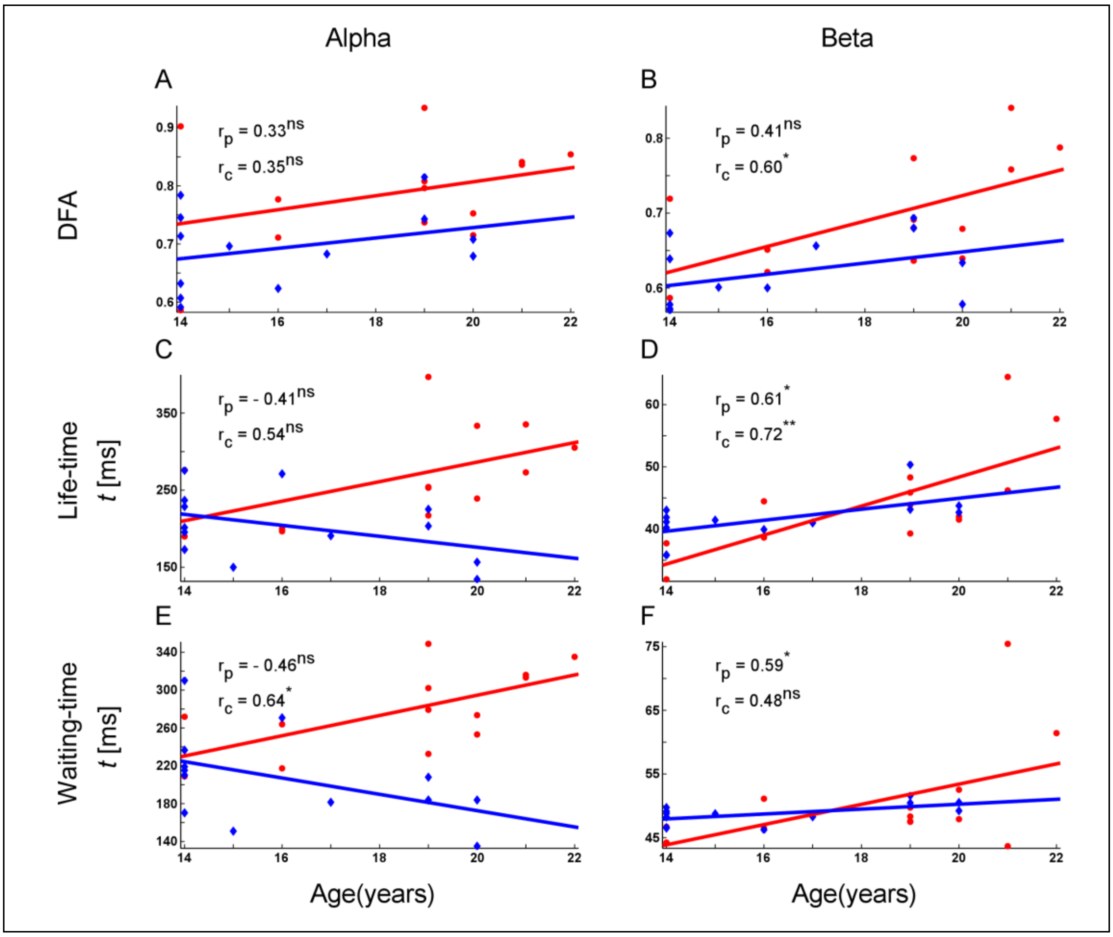

Fig.S2 The Pearson correlation coefficients between the age and DFA exponent (A and B), the age and life-time biomarker (C and D), and the age and waiting-time biomarker (E and F) of the alpha and beta bands for the patients and controls respectively. The blue diamonds indicate the patients, and the red dots indicate the controls. Among all of the electrodes, the electrode with the maximal correlation coefficient was selected to plot the scatter plot for the alpha (A, C and E) and beta (B, D, and F) bands. As can be seen above, some correlation coefficients were significant, others were not significant, but all of the correlation coefficients were not significant after the FDR correction. p: patients, c: controls, ns: no significance, *: $p < 0.05$, **: $p < 0.01$.
